# Multimodal evidence for delayed threat extinction learning in adolescence and young adulthood

**DOI:** 10.1038/s41598-019-44150-1

**Published:** 2019-05-23

**Authors:** Jayne Morriss, Anastasia Christakou, Carien M. van Reekum

**Affiliations:** Centre for Integrative Neuroscience and Neurodynamics School of Psychology and Clinical Language Sciences University of Reading, Reading, UK

**Keywords:** Motivation, Human behaviour

## Abstract

Previous research in rodents and humans points to an evolutionarily conserved profile of blunted threat extinction learning during adolescence, underpinned by brain structures such as the amygdala and medial prefrontal cortex (mPFC). In this study, we examine age-related effects on the function and structural connectivity of this system in threat extinction learning in adolescence and young adulthood. Younger age was associated with greater amygdala activity and later engagement of the mPFC to learned threat cues as compared to safety cues. Furthermore, greater structural integrity of the uncinate fasciculus, a white matter tract that connects the amygdala and mPFC, mediated the relationship between age and mPFC engagement during extinction learning. These findings suggest that age-related changes in the structure and function of amygdala-mPFC circuitry may underlie the protracted maturation of threat regulatory processes.

## Introduction

The ability to discriminate between cues that signal threat and safety is crucial to an organism’s wellbeing. This ability not only supports escape and avoidance defense mechanisms, but also prevents disproportionate reactivity, ensuring long term protection against chronic stress and psychopathology^1^. Brain circuitry at the core of threat discrimination learning includes areas within the amygdala and medial prefrontal cortex (mPFC)^1^. During threat acquisition, a neutral cue comes to be associated with an aversive outcome (conditioned stimulus (CS)), which leads to greater amygdala activity to the cue^2^. During threat extinction training, the CS is presented without the aversive outcome, which results in reduced amygdala responses to the CS over time^3^, with the mPFC playing a critical role^1,4,5^.

Recent research with rodents and humans has shown distinctive developmental profiles of blunted threat extinction recall in adolescents^6–11^, relative to children and adults. For example, adolescents continue to show signs of defensive responding to previously learned threat cues (freezing in rodents and elevated skin conductance in humans), suggesting an age-specific relative dysregulation of the process that updates threat associations as safe^7,10–13^. Immunohistochemical evidence points to reduced synaptic plasticity in areas of the mPFC after extinction recall in adolescent rodents^7,10^. Despite these advances, direct evidence regarding the neural circuitry underlying blunted threat extinction learning in adolescent humans is limited. Two studies have reported no significant adolescent-related effects in the amygdala or mPFC during extinction learning^11,14^. However, during extinction recall, adolescents compared to adults show reduced mPFC activation, and anxious youth, compared to non-anxious youth show different patterns of task-related functional coupling between the amygdala-mPFC^15^.

Developmental alterations in amygdala-prefrontal cortical interactions are thought to be responsible for threat extinction differences seen in adolescence^16^. Research has demonstrated amygdala-prefrontal connectivity to change with age at rest^17^ and during the processing of affective information^18^. Moreover, the amygdala and mPFC undergo substantial structural change during development: the amygdala shows linear increases in grey matter across late childhood and adolescence, while the mPFC is characterised by quadratic changes, with substantial grey matter growth across childhood followed by grey matter pruning across adolescence^19–21^. Furthermore, the uncinate fasciculus, a white matter tract that connects the amygdala and mPFC, undergoes protracted growth across adolescence and into early adulthood^22,23^. Structural abnormalities, such as reduced grey matter volume in the mPFC and weaker integrity of the uncinate fasciculus, have been reported in adults with anxious temperament and anxiety disorders^24–28^. While the number of studies examining structural changes in anxious vs. non-anxious adolescents is low, there is some evidence of similar structural abnormalities in ventral portions of the prefrontal cortex and the uncinate fasciculus for anxious youth^29–34^. Findings in the amygdala regions for anxious vs. non-anxious adolescents are less clear, with some studies reporting larger volumes in the right and left amygdala, specifically the basolateral amygdala^35,36^, smaller volumes in the left amygdala^31,37,38^ or no difference in amygdala volume^39^.

The functional and structural evidence outlined above, combined with the frequently reported emergence of phobic anxiety disorders during late childhood and adolescence^40^, suggest this period to be an important and vulnerable window of threat extinction circuitry development. Therefore, examining the function and structure of threat extinction circuitry in healthy adolescents will further our understanding of how threat extinction learning processes develop, which may be relevant for future models of development in relation to anxiety disorder pathology.

In the current study we examined: (1) age-dependent changes in recruitment of the amygdala and mPFC during threat extinction learning, and (2) the age-dependent relationship between structure and function in the amygdala and mPFC. We used event-related functional magnetic resonance imaging (fMRI) of a cued threat conditioning paradigm, where an aversive sound served as an unconditioned stimulus and visual shapes as conditioned stimuli^41,42^. In vulnerable populations, such as children and adolescents, sound stimuli are recommended over mild electric shocks in threat conditioning paradigms^7,8,13,43^. CS-US pairings were 100% reinforced during threat acquisition to match the reinforcement schedule typically employed in the developmental animal literature^6–10^. We have previously shown the same experimental design to successfully evoke concomitant threat extinction psychophysiology and brain activation in adult populations^41,44^, similar to other labs using 100% reinforcement^45^. In addition, we collected structural magnetic resonance imaging (sMRI) and diffusion tensor imaging (DTI) data to measure structural changes in grey matter in the mPFC and amygdala, and in white matter in the uncinate fasciculus respectively.

We hypothesized that younger age (adolescents) would be associated with relatively blunted threat extinction learning. We expected this effect to be indexed by greater amygdala activation, reduced mPFC recruitment and elevated self-reported uneasiness ratings and skin conductance responses to the learned threat vs. safety cues during threat extinction learning. Furthermore, we hypothesized that the pattern of functional activation in younger aged participants would, at least in part, be associated with higher grey matter in the mPFC, lower grey matter in the amygdala, and weaker structural integrity in the uncinate fasciculus that connects the two regions.

## Results

Three participants did not complete the scanning procedure and three participants were removed due to excessive head movements (mean relative displacement over 3 mm), leaving forty-nine participants for analysis (*M* age = 18.70 yrs, *SD* age = 3.64 yrs, range = 12–28 yrs; 31 females & 18 males; Ethnicity: 44 identified as white, 3 as mixed, 1 as Asian, 1 black). The ratio of females and males was similar across age (Females under 18 yrs: *M* age = 15.31 yrs, *SD* age = 1.42, *n* = 15; Males under 18 yrs: *M* age = 15.98 yrs, *SD* age = 1.74, *n* = 8; Females over 18 yrs: *M* age = 21.44 yrs, *SD* age = 2.97, *n* = 15; Males over 18 yrs: *M* age = 21.62 yrs, *SD* age = 1.67, *n *= 11).

### Ratings

All participants rated the sound stimulus as aversive (*M* = 2.96, *SD* = 1.17) and moderately arousing (*M* = 5.10, *SD* = 1.94). Sound arousal ratings were negatively correlated with age, *r*(47) = −0.286, *p* = 0.047 such that increasing age was associated with lower rated arousal. Sound valence ratings did not correlate with age, *r*(47) = −0.141, *p* = 0.333.

During extinction, participants reported feeling significantly more uneasy during the CS+(*M* = 1.04, *SD* = 1.16) compared to the CS− (*M* = 0.92, *SD* = 1.14) trials across the extinction learning phase, *F*(1,47) = 5.094, *p* = 0.029, η^2^ = 0.098. In addition, participants also reported feeling more uneasy at the start of extinction, compared to the end of extinction *F*(1,47) = 6.875, *p* = 0.012, η^2^ = 0.128. Contrary to predictions, there was no interaction of Stimulus × Time, *F*(1,47) = 1.004, *p* = 0.322 for the uneasiness ratings.

Results revealed no age differences for uneasiness ratings in any of the experimental phases, max *F* = 0.815.

### SCR magnitude

During extinction learning, participants displayed larger SCR magnitude to the CS+ (*M* = 0.13, *SD* = 0.28) compared to the CS− (*M* = −0.07, *SD* = 0.29), *F*(1,34) = 7.248, *p* = 0.011, η^2^ = 0.17. No significant interactions between Stimulus and Age or Time were observed during extinction, max *F* = 3.092.

### fMRI

**Figure 1:**
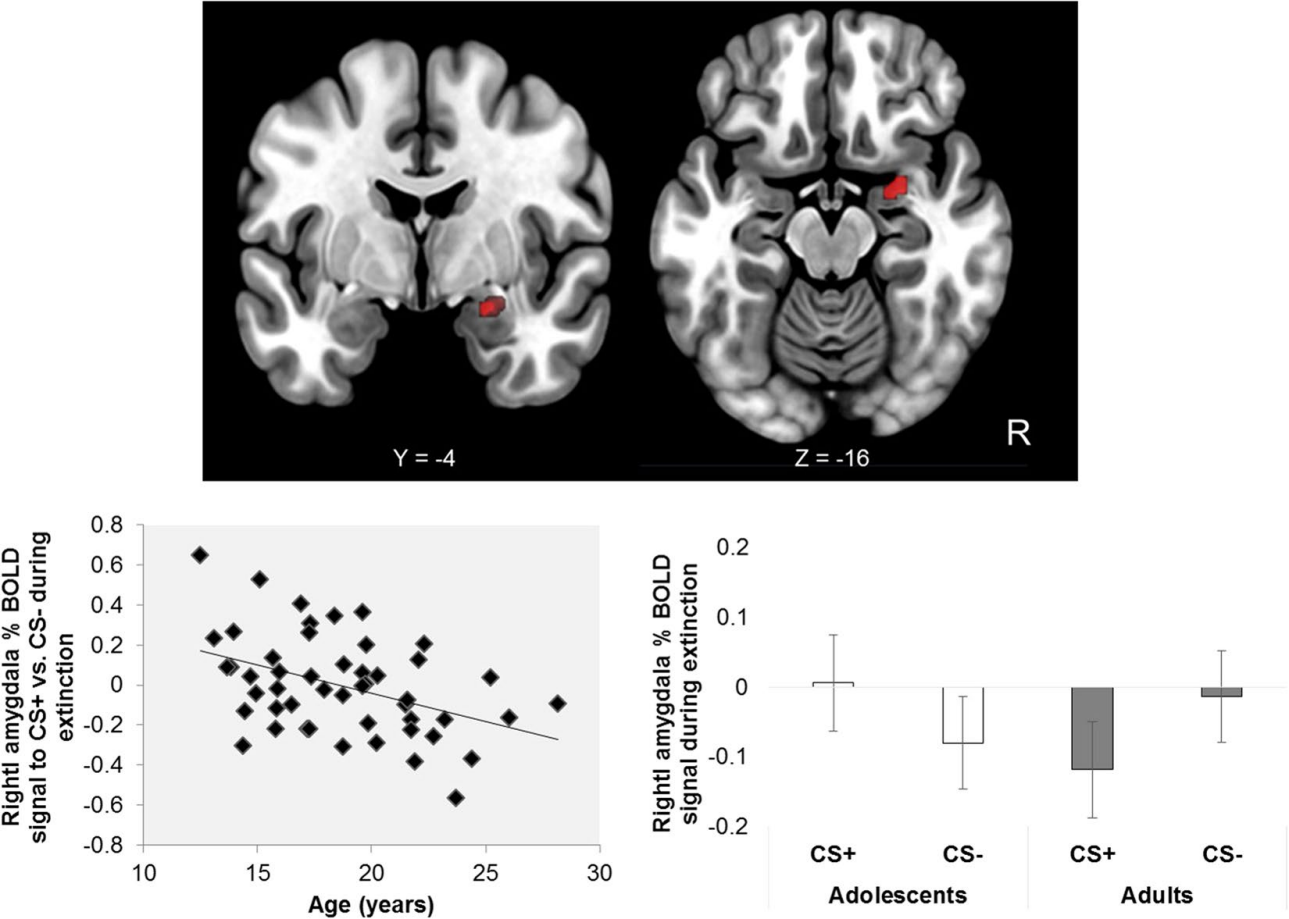
Top panel: Right amygdala activation to CS+ > CS− × decreasing age. Bottom left panel: Younger age is significantly associated with greater right amygdala activation to the CS+ vs. CS− across the threat extinction phase. Bottom right panel: Right amygdala activation to CS+ and CS− during extinction displayed by groupings of adolescents (1 stdev below the mean: 15 yrs of age) and adults (1 stdev above the mean: 22 yrs of age). Coordinates in the top panel are in MNI space. R = right.

**Figure 2:**
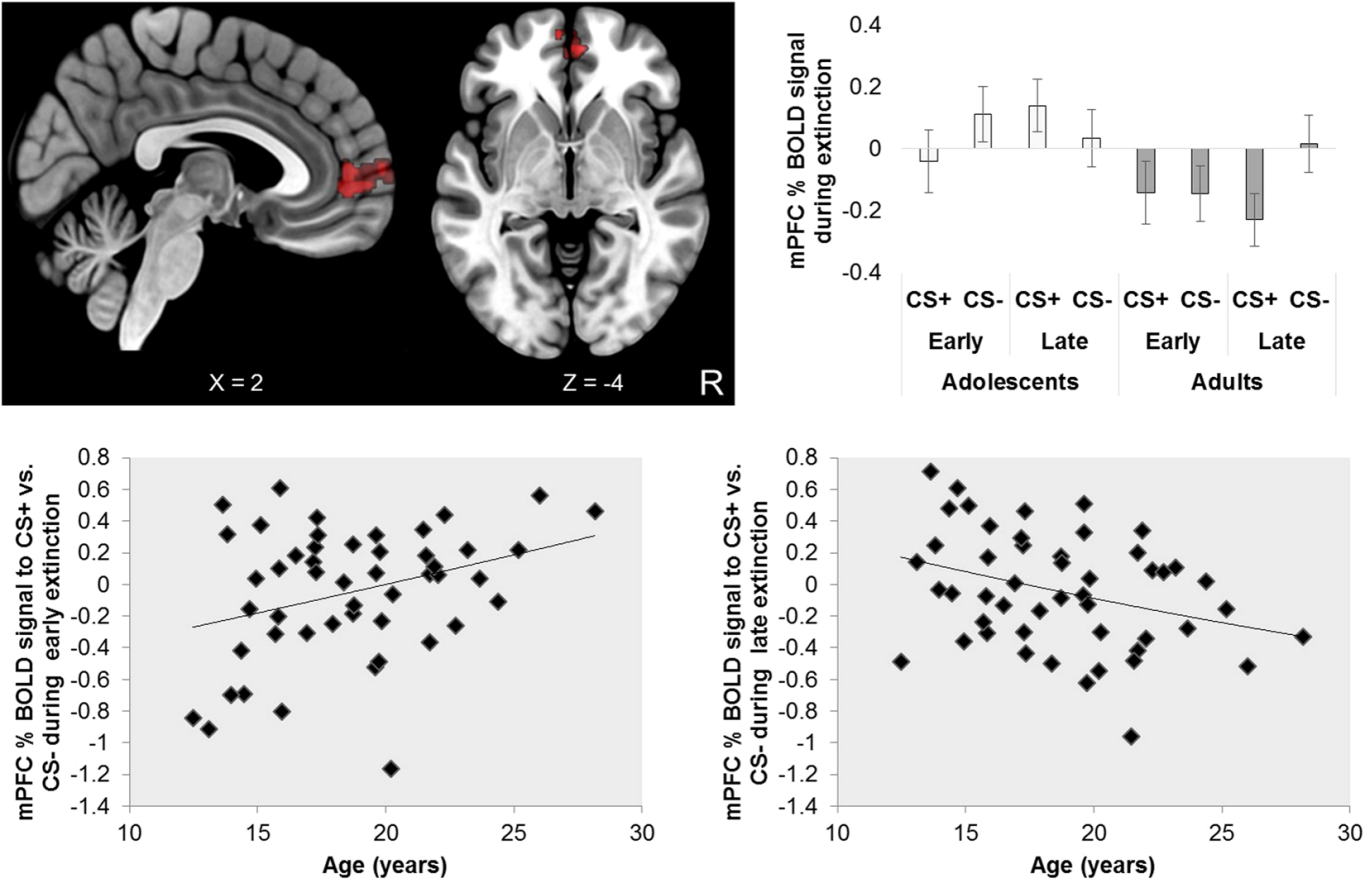
Top left panel: mPFC activation to CS+ > CS− early − CS+ > CS− late × increasing age. Bottom left and right panels: Older age is significantly associated with greater mPFC activity to the CS+ vs. CS− during early threat extinction, whilst younger age is significantly associated with greater mPFC activity to the CS+ vs. CS− during late threat extinction. Top right panel: mPFC activation to CS+ and CS− during extinction displayed by groupings of adolescents (1 stdev below the mean: 15 yrs of age) and adults (1 stdev above the mean: 22 yrs of age). Coordinates in the top panel are in MNI space. R = right.

A full list of details of clusters of activation in a-priori regions of interest and whole-brain corrected for key contrasts is provided in Table 1. During threat extinction learning younger age was associated with greater right amygdala activity to the CS+ vs. CS− (significant Stimulus × Age interaction) across the entire extinction learning phase (see Fig. 1 and Table 1). The analysis of extinction over time (Stimulus × Time × Age interaction), showed increased recruitment of the mPFC to CS+ vs. CS− in the early trials for older participants and in the late trials for younger participants (see Fig. 2 and Table 1).

**Table 1:** Regional activation patterns in response to extinction.

Contrast	Brain region	Voxels	Max Z	Location of max Z		
				x	y	z
**Age effects**						
CS+ > CS− × decreasing age	A-priori ROI/SVC: right amygdala	20	3.1	26	0	−16
CS+ > CS− × decreasing age	R putamen, R amygdala, R hippocampus	281	3.28	20	−90	32
CS+ > CS− × decreasing age	R lateral occipital cortex, R cuneal cortex	271	3.6	14	−2	−22
CS+ > CS− × increasing age	L postcentral gyrus	423	3.87	−54	−22	50
CS+ > CS− early − CS+ > CS− late × increasing age	A-priori ROI/SVC: medial frontal cortex, paracingulate gyrus, frontal pole	174	3.89	0	66	10
CS+ > CS− early - CS+ > CS− late × increasing age	L frontal pole	313	3.92	−22	42	50
**Main effects**						
CS − CS+	Posterior cingulate, precuneus cortex, L intracalcarine cortex, L lingual gyrus	1365	4	−24	−42	−14
CS − CS+	L postcentral gyrus, L precentral gyrus	502	4.74	−48	−14	48
CS − CS+	L postcentral gyrus, L precentral gyrus	392	3.38	−24	−52	74
CS − CS+	R lateral occipital cortex	387	4.35	42	−64	26
CS − CS+	L lateral occipital cortex	290	3.55	−42	−76	40
CS − CS+	L lingual gyrus	274	3.85	−22	−60	−14
CS − CS+	R postcentral gyrus, R precentral gyrus	256	4.22	40	−34	32
CS+ > CS− early − CS+ > CS− late	A-priori ROI/SVC: left ortibal frontal cortex	200	3.81	−34	34	−14
CS+ > CS− early − CS+ > CS− late	L occitpital pole	383	5.02	28	−96	−4
CS+ > CS− early − CS+ > CS− late	R occipital pole	277	3.86	−34	−98	−4
CS− > CS+early − CS− > CS+late	R postcentral gyrus, R supramarginal gyrus	419	4.01	52	−20	36
CS− > CS+early − CS− > CS+late	L postcentral gyrus, L supramarginal gyrus	378	3.75	−50	−28	38
CS− > CS+early − CS− > CS+late	L lingual gyrus	359	4.27	−16	−68	−8
CS− > CS+early − CS− > CS+late	L postcentral gyrus, L superior parietal lobule	356	3.78	−30	−44	50
CS− > CS+early − CS− > CS+late	R superior parietal lobule	337	3.42	28	−50	68

Note: Corrected cluster for multiple comparisons at p < 0.05. Location of cluster’s maximum Z are in MNI space. Voxel size is 2 × 2 × 2 mm. R = right; L = left. SVC = small volume corrected.

### Grey matter probability in the amygdala and mPFC

As mentioned in the Methods section, we extracted grey matter probability from the fMRI clusters identified in a-priori regions of interest and depicted in Figs 1 and 2, i.e. right amygdala and mPFC.

#### Age-structure relationships

As expected, we found a significant negative correlation between mPFC grey matter probability and age, *r*(47) = −0.436, *p* = 0.002. No significant correlation between the right amygdala and age was observed, *r*(47) = 0.139, *p* = 0.340.

#### Structure-function relationships

Grey matter probability in the mPFC significantly predicted the mPFC activation difference score for extinction (CS+ vs. CS −_Early_ − CS+ vs. CS− _Late_), *r*(47) = −0.342, *p* = 0.016. We found no significant relationships between grey matter probability in the mPFC or right amygdala with right amygdala activation during extinction, *r*(47) = 0.018, *p* = 0.904; *r*(47) = −0.167, *p* = 0.250.

#### Mediation of age-function by structure

Grey matter probability in the mPFC did not significantly mediate the relationship between age and mPFC activity during extinction learning, *t* = 1.103, *p* = 0.275.

### White matter integrity of the uncinate fasciculus

#### Age-structure relationships

In line with predictions, we found positive correlations with age and structural integrity of the bilateral uncinate fasciculus (the left and right uncinate fasciculus FA values significantly correlated, *r*(47) = 0.73, *p* < 0.001, thus we collapsed across hemisphere), *r*(47) = 0.30, *p* = 0.035, suggesting increased white matter integrity of this tract with advancing age.

#### Structure-function relationships

Greater structural integrity of the bilateral uncinate fasciculus predicted the mPFC activity difference score for extinction learning (CS+ vs. CS−_Early_ −CS+ vs. CS− _Late_), *r*(47) = 0.463, *p* = 0.001. As highlighted in the Introduction, white matter develops across the brain during adolescence. The bilateral uncinate fasiculus significantly predicted mPFC activity during extinction learning (second step: Δ*R*^2^ = 0.173, *F*(1,45) = 10.367, *p = *0.002), over and above the forceps minor and corticospinal tract (first step: *R*^2^ = 0.078, *F*(2,46) = 1.933, *p* = 0.156).

Structural integrity of the bilateral uncinate fasciculus was not significantly associated with right amygdala activity for CS+ vs. CS− during extinction learning, *r*(47) = −0.066, *p* = 0.655.

#### Mediation of age-function by structure

**Figure 3:**
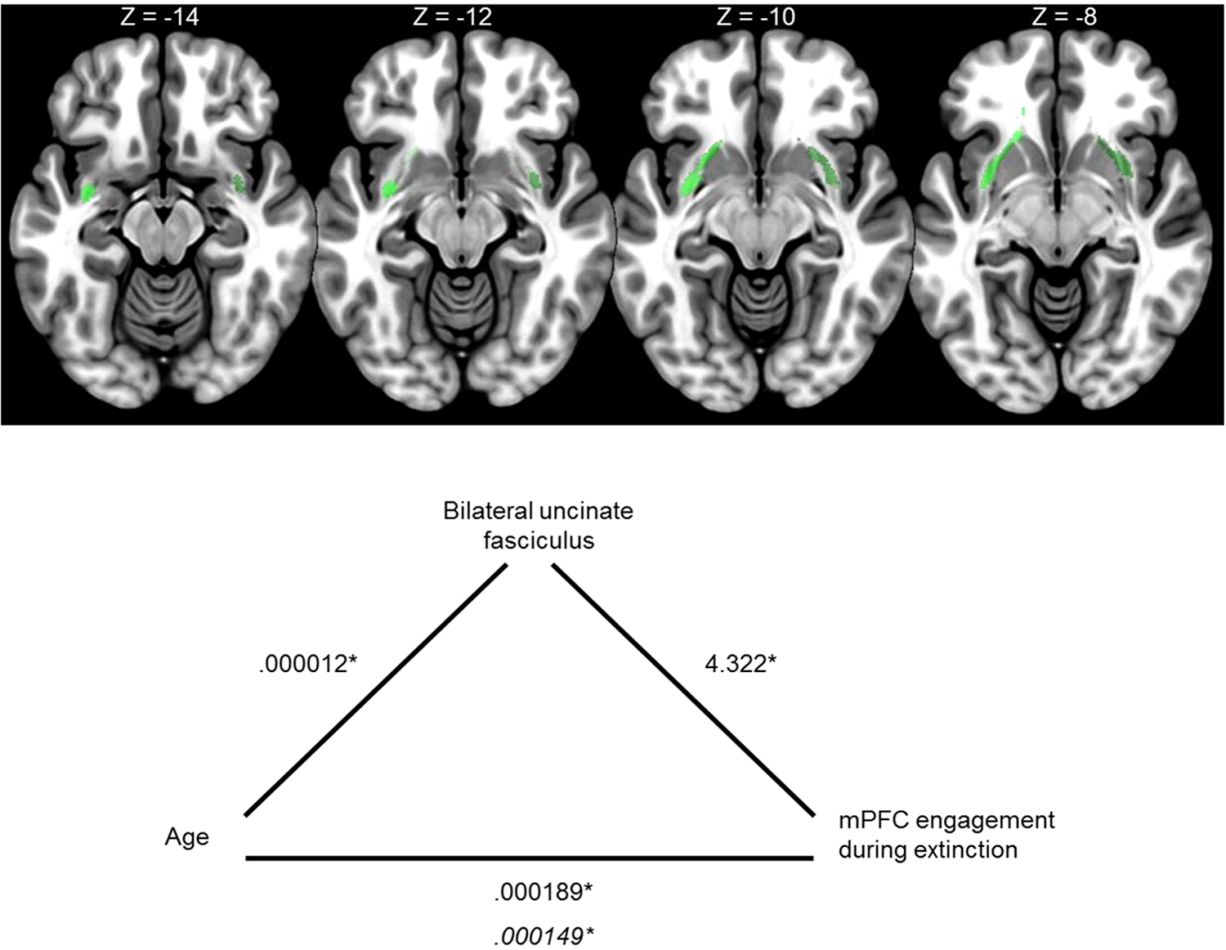
Top panel: Bilateral uncinate fasciculus brain masks. Bottom panel: Greater structural integrity of the unincate fasciculus mediated the relationship between age and mPFC modulation during extinction (difference score: CS+ vs. CS −Early − CS+ vs. CS− Late). Coordinates in the top panel are in MNI space. Values in the bottom panel represent the unstandardized coefficients from linear regression models. Italicized values represent the unstandardized coefficient for age and mPFC engagement after adjustment for the uncintate fasciculus mediator. *p < 0.05.

The structural integrity of the bilateral uncinate fasciculus significantly mediated the relationship between age and mPFC activity during extinction learning (difference score: CS+ vs. CS −_Early_ − CS+ vs. CS− _Late_), *t* = 2.695, *p* = 0.009 (see Fig. 3). In the mediation model, after adjustment for the bilateral uncinate fasciculus, age continued to predict mPFC activity but to a lesser extent (see Fig. 3).

## Discussion

In the current study, we show age-related changes in amygdala and mPFC recruitment during threat extinction learning. Our data suggest that adolescence, relative to adulthood, is associated with later recruitment of the mPFC and greater engagement of the amygdala to threat cues compared to safety cues. Furthermore, the relationship between age and later recruitment of the mPFC during extinction learning was mediated in part by the structural integrity of the uncinate fasciculus, a white matter tract connecting the amygdala and mPFC. These data contribute to the growing literature on threat extinction during development^6,8–11,13,46,47^.

Across extinction learning participants displayed greater uneasiness ratings and larger SCR magnitude to CS+ vs. CS− trials, suggesting effects of conditioning^48^. However, neither the uneasiness ratings nor SCR magnitude significantly varied with age. This is at odds with previous rodent and human research that has shown exaggerated defensive responding in adolescents^8,10,12,13,46,49^. In recent fMRI experiments no significant effect of age has been observed on SCR magnitude during extinction learning^11,50^. The lack of age-related SCR results in the current study and previous fMRI studies in adolescence may be due to: (1) participants lying down versus sitting which substantially reduces SCR activity and, (2) reduced SCR signal quality due to the fMRI scanner. Notably, younger age, relative to older age was associated with higher ratings of arousal to the US (i.e. aversive human scream), providing some evidence of greater emotional expression in adolescents.

During threat extinction learning, younger age was characterised by increased amygdala activity to threat vs. safety cues. Furthermore, younger age was associated with later recruitment of the mPFC to threat vs. safety cues during extinction learning, in line with previous rodent work showing alterations in prefrontal activity in adolescence after extinction recall^8,10^. Notably, a few fMRI studies did not observe significant age-related effects during threat extinction learning^11,14^. The difference in findings between the studies may be due to the age range used. In the current study the age ranged between 13–28 years, whilst in the previous studies the age range was truncated, as the adolescents were 14 years old^14^ or between 14–16 years old^11^. Despite this, the current study suggests that adolescents, relative to younger adults, exhibit reduced top-down modulation in threat extinction circuitry during learning.

Our data also show typical structural maturation in threat extinction circuitry. In line with prior structural work^19–22,51–53^, grey matter in the mPFC decreased with age, while white matter in the uncinate fasciculus increased with age. Grey matter probability in the mPFC and white matter integrity of the uncinate fasciculus were associated with age-dependent changes in mPFC responding during threat extinction learning. Grey matter in the amygdala was not correlated with amygdala BOLD response during extinction learning. However, only the uncinate fasiculus was found to mediate the relationship between age and mPFC responding during threat extinction learning. After adjustment for the contribution of the uncinate fasciculus in the mediation model, age continued to predict mPFC activity but to a lesser extent. These results suggest that both chronological age and structural changes in the uncinate fasciculus contribute to mPFC engagement during extinction.

Notably, weaker structural integrity of the uncinate fasciculus has been found in populations with anxious temperament^24–27,32^, suggesting that this tract may play a crucial role in anxiety aetiology. The uncinate fasciculus has a protracted growth, as it continues to develop until the third decade of life, unlike the expedited grey matter pruning in regions such as the mPFC. On this basis, following the development of the uncinate fasciculus may be particularly promising for neurodevelopmental researchers and clinicians. Future work with longitudinal designs may be able to further elucidate the relationship between threat extinction ability, white matter integrity in the uncinate fasciculus and anxiety disorder vulnerability by tracking the extent of malleability within the uncinate fasciculus across adolescence and into early adulthood. From this we may be able to identify whether anxious individuals have a different developmental trajectory of the uncinate faciculus and when it may be best to attempt treatment or intervention.

The current study had a number of limitations that will be addressed here. Firstly, it is difficult to infer the extent to which development impacts the function of threat extinction circuitry in the current study, given that it was cross sectional. Longitudinal studies are needed to assess the extent to which any age-related difference in mechanisms such as the ones reported here hold within-subjects. Secondly, the design specifics of the current study should be further addressed in future research to assess the robustness and generalizability of the extinction learning findings reported here. For example, we used a 100% reinforcement schedule, which made it difficult to assess age-related effects on the conditioned response during acquisition (see supplementary material). Moreover, in this study we used a fast event-related design, which is not optimal for conducting connectivity analyses (for additional psychophysiological interaction analyses see supplementary material). Lastly, to be more comparable with animal research, the study would have benefitted from using an extinction learning phase 24 hours after acquisition.

To conclude, we show age-related effects on threat extinction circuitry during adolescence. In our sample, younger age was associated with exaggerated amygdala responses to learned threat vs. safety cues during threat extinction learning. In addition, younger age was associated with reduced mPFC activity during early extinction learning. The relationship between age and delayed recruitment of the mPFC was mediated by the structural integrity of the uncinate fasciculus, a white matter tract connecting the amygdala and mPFC. Importantly, these results highlight the need for further examination of structural and functional changes in amygdala-mPFC circuitry during adolescence, particularly with respect to risk of anxiety disorder development^54,55^.

## Methods

### Participants

55 right-handed volunteers took part in this study (*M* age = 17.75 yrs, *SD* age = 3.65 yrs, range = 13–28 yrs; Sex: 35 females & 20 males; Ethnicity: 49 identified as white, 3 as mixed, 2 as Asian, 1 black). All participants had normal or corrected to normal vision/hearing. Participants were excluded if they were left-handed or had fMRI contraindications. No other exclusion criteria were applied. The sample size was determined using sample estimates for group fMRI based on 80% power^56^. From this 20 participants per group (adolescent/adults) was recommended. We over sampled due to: (1) examining age as a continuous predictor, and (2) the developmental population of interest, where we expected participant attrition from claustrophobia and excessive head movement.

Adult participants provided written informed consent, adolescent participants provided written informed assent and the parent/guardian provided informed consent, and received a picture of their brain and £20 for their participation. The procedure was approved by the University of Reading Ethics Committee. The methods were carried out in accordance with the relevant guidelines and regulation.

### Procedure

The procedure was near identical to a previous study^41,42^. First, participants (and parents/guardians) completed consent forms as an agreement to take part in the study. Second, a hearing test was performed with an audiometer (all participants fell within the normative range of 500–8000 Hz, below 30 dB). Third, participants completed a battery of cognitive tasks (Switch Task, Stroop Task, Letter Memory Task, data not reported here) and questionnaires on a computer outside of the scanner (BISBAS, Trait Anxiety, Intolerance of Uncertainty, data not reported here). Next, participants were taken to the MRI unit. We used a conditioning task inside the scanner, whilst concurrently recording ratings and skin conductance (as well as pupil dilation, data not reported here). After scanning, participants rated sound stimuli presented in the scanner and completed another computerised task (not reported here).

### Conditioning task

The conditioning task was identical to those used in previous studies^41,42,44^. The task was designed using E-Prime 2.0 software (Psychology Software Tools Ltd, Pittsburgh, PA). Visual stimuli were presented through MRI-compatible VisualSystem head-coil mounted eye goggles (Nordic Neuro Lab, Bergen, Norway), which displayed stimuli at 60 Hz on an 800 × 600 pixel screen, with a field of view of 30° × 23°. Sound stimuli were presented through MRI-compatible AudioSystem headphones (Nordic Neuro Lab, Bergen, Norway).

Visual stimuli were light blue and yellow squares with 183 × 183 pixel dimensions that were matched for brightness. The aversive sound stimulus consisted of a threat inducing female scream from the International Affective Digitized Sound battery (IADS-2, sound number 277)^57^. We used Audacity 2.0.3 software (http://audacity.sourceforge.net/) to shorten the female scream to 1000 ms in length and to amplify the sound by 15 dB, resulting in a 90 dB (within +/−5 dB of 90 dB) sound. An audiometer was used before testing to standardize the sound volume across participants.

The conditioning task consisted of three learning phases that were presented in three separate blocks across the scan. (1) In acquisition, one of the squares was paired with the aversive 90 dB scream (CS+), whilst the other square was presented alone (CS−). (2) In extinction learning, no US sounds were presented. (3) During reacquisition, the CS+ square was paired with the sound 25% of the time, and the CS− remained alone. We do not report the acquisition phase as the CS+ is paired 100%, thus the CS+ is confounded with the US. We focus our reporting on the extinction learning phase.

The acquisition phase consisted of 24 trials (12 CS+, 12 CS−), the extinction learning phase of 32 trials (16 CS+, 16 CS−). Experimental trials within the conditioning task were pseudorandomized into an order, which resulted in no more than three presentations of the same stimulus in a row. Conditioning contingencies were counterbalanced, with half of the participants receiving the US with a blue square and the other half of participants receiving the US with a yellow square. Participants were instructed to attend and listen to the stimulus presentations, as well as respond to a rating scale that followed each trial. The rating scale asked how ‘uneasy’ the participant felt after each stimulus presentation, where the scale was 0 ‘not at all’–10 ‘extremely’. Participants used a hand-held MRI-compatible response box in their dominant right hand.

The presentation times of the acquisition phase were: 1500 ms square paired with a 1000 ms sound played 500 ms after the onset of a CS+ square, 3000–6450 ms blank screen, 4000 ms rating scale, and 1000–2500 ms blank screen. The presentation times were the same for the extinction phase, but the 1000 ms sound was omitted. To avoid predictability of stimulus on/offset, and to maximize design efficiency, we introduced jitter by randomly selecting the duration of the blank screens for each trial from the range indicated above.

### Sound stimulus rating

Participants rated the valence and arousal of the sound stimulus using 9 point Likert scales ranging from 1 to 9 (Valence: negative to positive; Arousal: calm to excited).

### Behavioral data scoring and reduction

Rating data were reduced for each subject by calculating their average responses for each experimental condition using the E-Data Aid tool in E-Prime (Psychology Software Tools Ltd, Pittsburgh, PA).

### Physiological acquisition and reduction

Skin conductance recordings were obtained using AD Instruments (AD Instruments Ltd, Chalgrove, Oxfordshire) hardware and software. An ML138 Bio Amp connected to an ML870 PowerLab Unit Model 8/30 amplified the electrodermal activity signal, which were digitized through a 16-bit A/D converter at 1000 Hz. Skin conductance was measured during the fMRI scanning with MRI-compatible MLT117F silver/silver chloride bipolar finger electrodes that were attached to the distal phalanges of the index and middle fingers of the left hand. A low constant-voltage AC excitation of 22mVrms at 75 Hz was passed through the electrodes, which were connected to a ML116 GSR Amp, and converted to DC before being digitized and stored.

Skin conductance responses (SCR) were scored when there was an increase of skin conductance level exceeding 0.03 microSiemens^58^. The amplitude of each response was scored as the difference between the onset and the maximum deflection prior to the signal flattening out or decreasing. SCR onsets and respective peaks were counted if the SCR onset was within 0.5–7 seconds following the CS onset. Trials with no discernible SCRs were scored as zero. The first trial of each experimental phase was excluded, to reduce contamination of averages from the orienting response typically seen at the start of a session. SCR amplitudes were square root transformed to reduce skew^58^ and z-scored to reduce individual differences in skin conductance responding due factors not of interest i.e. dryness of the skin^59^. SCR magnitudes were calculated from remaining trials by averaging SCR magnitude values and zeros for each condition. Thirteen subjects were removed from the SCR magnitude analysis because of non-responding (i.e. responding to none of the trials during extinction) leaving thirty-six subjects for analysis. The non-responders were only excluded from the SCR magnitude analysis and were included in analyses of other measures.

### MRI

The scanning protocol was identical to a previous experiment^41,42^. Participants were scanned with a 3 T Siemens Trio set up with a 12 channel head coil (Siemens Inc., Erlangen, Germany). Three T2*-weighted echo planar imaging (EPI) functional scans were acquired for each phase of the conditioning task consisting of 161, 208, and 380 volumes respectively (TR = 2000 ms, TE = 30 ms, flip angle = 90°, FOV = 192 × 192 mm, 3 × 3 mm voxels, slice thickness 3 mm with an interslice gap of 1 mm, 30 axial slices, interleaved acquisition).

Following completion of the functional scans, fieldmap and structural scans were acquired, which comprised of a high-resolution T1-weighted anatomical scan (MP-RAGE, TR = 2020 ms, TE = 2.52 ms, flip angle = 90°, FOV = 256 × 256 mm, 1 × 1 × 1 mm voxels, slice thickness 1 mm, sagittal slices), two fieldmaps (TR = 488 ms, TE 1 = 4.98 ms, TE 2 = 7.38 ms, flip angle = 60°, FOV = 256 × 256 mm, slice thickness 4 mm with an interslice gap of 4 mm, 30 axial slices) and diffusion weighted images (TR = 6800 ms, TE = 93 ms, flip angle = 60°, FOV = 192 × 192 mm, slice thickness 2 mm with an interslice gap of 2 mm, *b*-value = 1000, 64 axial slices, 30 diffusion gradients).

### fMRI analysis

FMRI analyses were carried out in Feat version 5.08 as part of FSL (FMRIB’s Software Library, www.fmrib.ox.ac.uk/fsl). Each brain was extracted from their respective T1-weighted image by using the FSL Brain Extraction Tool (BET)^60^. Distortion, slice timing and motion correction were applied to all EPI volumes using FUGUE and MCFLIRT tools^61^. Gaussian smoothing (FWHM 5 mm) and a 50 second high-pass temporal filter were applied.

A first-level GLM analysis was carried out for the extinction phase. Separate regressors were specified for the experimental conditions of primary interest (CS+/CS) by convolving a binary boxcar function with an ideal haemodynamic response (HR), which corresponded to the length of each trial (1500 ms). Regressors for the uneasiness rating period, six motion parameters (based on rotation and translation from 6 degrees of freedom) and any head movements between 1–3 mm were included to model out brain activity or movement artefacts that were unrelated to the conditions of interest.

We defined two main effect contrasts to reveal threat extinction-related activity (similar to previous work^41^). To examine temporal effects across extinction learning, we contrasted (CS+ vs. CS−)_Early_ > (CS+ vs. CS−)_Late_. We defined early extinction as the first eight trials for CS+ and CS− and the last eight trials for CS+ and CS−, similar to previous extinction research (for review see,^48^). We also examined the overall effect of CS+ vs. CS− during extinction. All contrasts were normalized and registered to MNI standard space using FLIRT^61^. Second-level GLM analysis consisted of regressors for the group mean and a linear regressor for demeaned age scores using FSL’s Ordinary Least Squares procedure with a cluster thresholding of *z* = 2.3 and a corrected *p* < 0.05 (additional permutation and psychophysiological interaction analyses were also conducted, see supplementary material).

We were specifically interested in the extent to which age would be associated with the BOLD response in the amygdala and mPFC for threat extinction learning. Therefore, we performed small volume corrections on the left amygdala, right amygdala and medial prefrontal cortex using cluster thresholding with a *z* = 2.3 and a corrected *p* < 0.05 on the age × extinction (CS+ vs. CS−) and age × extinction (CS+ vs. CS−)_Early_ > (CS+ vs. CS−)_Late_ contrast maps. We used anatomically defined masks from the Harvard-Oxford cortical and subcortical structural atlases in FSL^62^. We selected the left amygdala, right amygdala and prefrontal (including the subgenual anterior cingulate, medial and orbitofrontal portions, and excluding the lateral portion) cortical regions with a 50% probability threshold (see supplementary material).

### Grey matter probability in the amygdala and mPFC

Processing of structural images was performed in FSL. Firstly, structural images were brain-extracted using BET^60^. Secondly, structural images were segmented based on tissue-type using FMRIB’s Automated Segmentation Tool (FAST)^63^. Thirdly, any resulting clusters of age-related activation in the amygdala or mPFC identified from the fMRI analysis were transformed into structural space for each subject. Lastly, we extracted grey matter probability estimates from the amygdala and mPFC clusters from each subjects’ segmented structural image.

### White matter integrity of the uncinate fasciculus

Diffusion-weighted image processing in FSL included corrections for motion, eddy currents and inhomogeneities in the magnetic field. Then the tensor model was fitted using FDT (FMRIBS Diffusion Toolbox) in order to calculate FA values for each voxel, producing one FA image per subject. Voxels with FA values lower than 0.2 were removed. We created 25% probability masks of the left and right uncinate fasciculus the forceps minor and the corticospinal tract from the JHU white-matter tractography atlas^64^. The forceps minor and corticospinal tract served as control regions, similar to previous structure-function research^65^. All tract masks were transformed into diffusion space and applied to each subjects’ FA image, resulting in an FA value for each tract per subject.

### Statistical analyses

Main effects of conditioning and age in threat extinction were assessed by conducting a Stimulus (CS+, CS−) × Time (Early, Late) × Age (days) repeated measures ANCOVA on uneasiness ratings and SCR magnitude. Age was entered as a continuous predictor variable. The early part of extinction was defined as the first eight CS+ and CS− trials, and the last part of extinction was defined as the last eight CS+ and CS− trials. Interaction effects were followed up with pairwise comparison using least squared difference.

We correlated any resulting areas of age-related activation in the amygdala or mPFC identified from the fMRI analysis with grey matter probability estimates of those regions and FA values in the uncinate fasciculus. To examine whether the relationship between mPFC activation during extinction and FA values was specific to the uncinate fasciculus we conducted control analyses in the forceps minor and corticospinal tract.

Furthermore, if there were relationships between age and function/structure then we conducted follow up mediation analyses to assess whether structure mediated the relationship between age and functional engagement of the amygdala or mPFC. We used Freedman-Schatzkin mediation tests, where the difference in the regression coefficient between the unadjusted association between the independent variable and the dependent variable is compared with the regression coefficient for this association when it is adjusted for a potential intervening or mediating variable^66^.

## Supplementary information

Supplementary Material

## Data Availability

For access to the data please contact Dr. Jayne Morriss.
