# Supplementary material for: Multimodal evidence for delayed threat extinction learning in adolescence and young adulthood
Source: Sci Rep. 2019 May 23;9:7748. doi: 10.1038/s41598-019-44150-1 (PMC6533253; doi:10.1038/s41598-019-44150-1)
Supplement: Supplementary file 1 — Supplementary Material [file 41598_2019_44150_MOESM1_ESM.pdf]

Multimodal evidence for delayed threat extinction learning in adolescence and young adulthood.

Jayne Morriss[a*], Anastasia Christakou[a], and Carien M. van Reekum[a]

[a]Centre for Integrative Neuroscience and Neurodynamics

School of Psychology and Clinical Language Sciences

University of Reading

Reading

UK

*Correspondence:

Jayne Morriss

Centre for Integrative Neuroscience and Neurodynamics

School of Psychology and Clinical Language Sciences

University of Reading

Earley Gate, Whiteknights Campus

RG6 6AH Reading

United Kingdom

j.e.morriss@reading.ac.uk

Supplementary Material

fMRI Masks

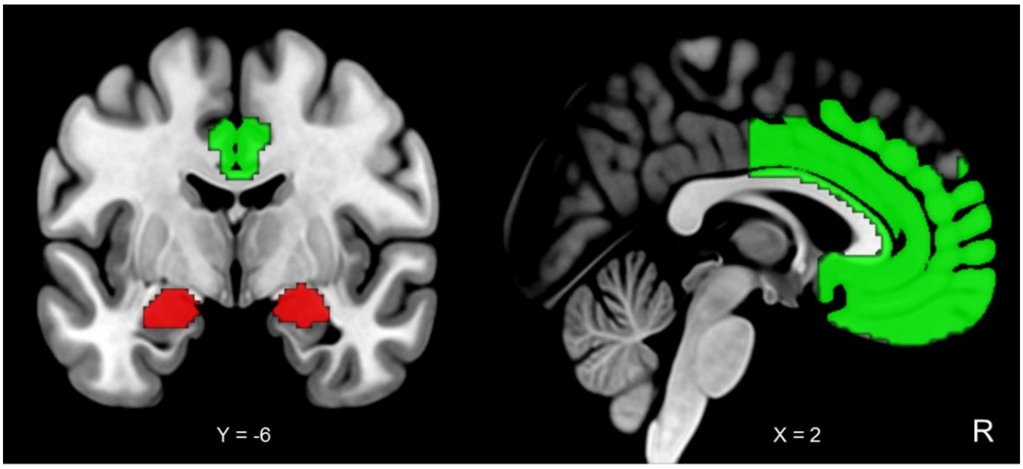

Supplementary Figure 1. Masks used for the region of interest analysis. Red = Left and right amygdala. Green = medial prefrontal cortex. Coordinates are in MNI space. R=right.

fMRI data using permutation testing

The default value for the cluster forming threshold is z = 2.3 in the FSL package for fMRI data. However, the use of cluster forming thresholds with parametric statistics has been argued to lead to greater chance of false positives. Non-parametric approaches have been demonstrated as robust and valid, as they are based upon actual NULL data rather than a model of such data (Eklund, Nichols and Knutsson, 2016). Therefore, to test the robustness of our results, we reran our analyses using a non-parametric permutation test in FSL (i.e. Randomise with Threshold Free Cluster Enhancement (TFCE) using 5000 samples and corrected at $p < 0.05$ based on the number of voxels within each mask).

The results from the permutation test were similar to our original analysis. The stimulus x age interaction for the amygdala survived multiple comparisons. The

stimulus x time x age interaction for the mPFC did not survive multiple comparisons, however was still observable under threshold. The difference in strength of the findings between the amygdala and mPFC may be reflected by the size of the masks used for small volume correction. We believe that the results from the original analysis are genuine, given the similarity between the original analysis and the permutation test, as well as activation patterns of the mPFC found in previous extinction literature using fMRI (Fullana et al., 2018).

Psychophysiological interaction analysis

We used the right amygdala and medial prefrontal cortex as seed regions for the extinction phase. Second-level GLM analysis consisted of regressors for the group mean and a linear regressor for demeaned age and uncinate fasciculus scores using FSL's Ordinary Least Squares procedure with a cluster thresholding of $z = 2.3$ and a corrected $p < 0.05$. We did not observe any significant clusters for the right amgydala or medial prefrontal cortex seeds. The results are the same if we include both regressors in the model (demeaned age and uncinate fasciculus scores) or enter the two regressors separately in the model. The lack of connectivity results may have been due to our event-related design, which is not optimal for PPI analysis.

Correspondence between self-reported ratings and structure/function measures

We used correlations to assess whether there was any correspondence between self-reported ratings and structure/function measures. The uneasiness rating difference score (CS+ - CS-) for extinction did not significantly correlate with percent BOLD signal difference scores (CS+ - CS-) for the amygdala or mPFC, or structural

measures (i.e. amygdala and mPFC grey matter probability, fractional anisotropy for the uncinate fasciculus), p’s > .1.

Correspondence between self-reported pubertal stage and structure/function/psychophysiology measures

We collected data on pubertal stage from the adolescent participants (under 18 yrs) using the Pubertal Development Scale (PDS; Petersen, Crockett, Richards, & Boxer, 1988). To rule out any effects of pubertal stage, we used correlations to assess whether there was any correspondence between self-reported puberty stage and structure/function/psychophysiology measures. Pubertal stage was positively correlated with age in our sample, r(22) = .63, p = .001. Pubertal stage was not significantly correlated with percent BOLD signal difference scores (CS+ - CS-) for the amygdala or mPFC, difference scores (CS+ - CS-) for skin conductance, or structural measures (i.e. amygdala and mPFC grey matter probability, fractional anisotropy for the uncinate fasciculus), p’s > .1.

Learning assessment during acquisition

Age differences in acquisition could provide an alternative explanation for the age differences observed in extinction. To rule this out, we examined the conditioned response during acquisition using pupil dilation, as this was the only measure which could be analysed without the confound of the US.

Pupil dilation was sampled at 60 Hz through a built-in infrared camera on the head-coil mounted eye goggles (Nordic Neuro Lab, Bergen, Norway). Pupil dilation data for each trial in: (1) acquisition was averaged from 0-500ms after stimulus onset to assess conditioning. This was to avoid contamination from the onset of the US. (2)

Extinction was averaged from 0-5000 ms after stimulus onset. All data were baseline corrected by subtracting 1000 ms preceding each stimulus onset from a blank screen. Trials were averaged across their respective condition for each subject.

Main effects of conditioning and age in threat acquisition were assessed by conducting a Condition Stimulus (CS+, CS-) x Age (days) repeated measures ANCOVA on pupil dilation. Main effects of conditioning and age in threat extinction learning were assessed by conducting a Condition Stimulus (CS+, CS-) x Time (Early, Late) x Age (days) repeated measures ANCOVA on pupil dilation. Age was entered as a continuous predictor variable. The early part of extinction was defined as the first eight CS+ and CS- trials, and the last part of extinction was defined as the last eight CS+ and CS- trials. Interaction effects were followed up with pairwise comparison using least squared difference.

Pupil dilation data could not be collected from thirteen participants due to problems calibrating the goggles, leaving thirty-seven subjects with usable pupil dilation data.

During acquisition, pupil dilation was larger to the CS+ vs. CS-, before US presentation, suggesting participants learned the CS-US contingency, $F(1,35) = 4.520$, $p = .041$, $\eta 2 = .114$ (see supplementary Fig 2). Age was not significantly associated with the difference between the CS+ vs. CS-, $F(1,35) = .007$, $p = .934$, $\eta 2 < .001$. No significant main effects or interactions with age were found for extinction, max $F = 2.949$, $p = .095$ (see supplementary Fig 2).’

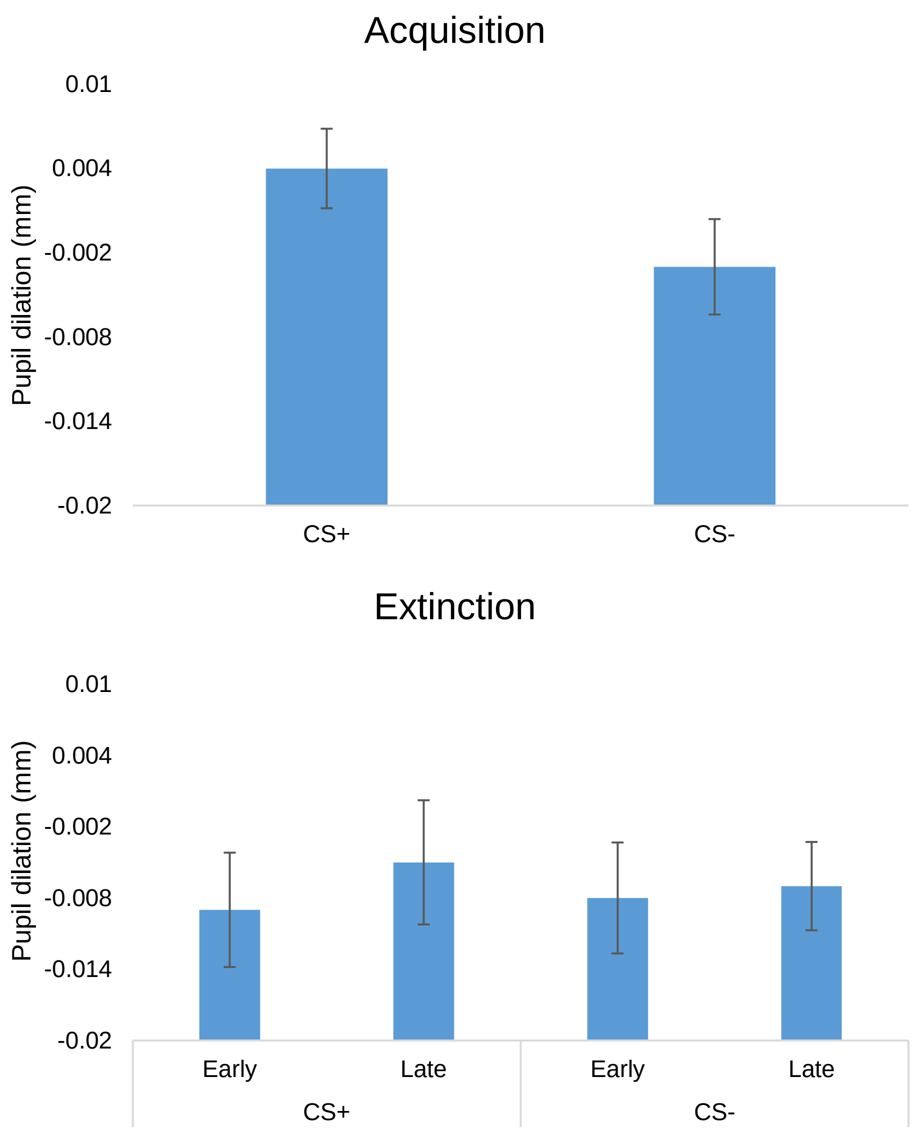

*Supplementary Figure 2***.** Pupil dilation response during acquisition and extinction. Pupil dilation measured in millimeters.

References

Eklund, A., Nichols, T. E., & Knutsson, H. (2016). Cluster failure: why fMRI inferences for spatial extent have inflated false-positive rates. *Proceedings of the National Academy of Sciences*, 113(28), 7900-7905.

Fullana, M. A., Albajes-Eizagirre, A., Soriano-Mas, C., Vervliet, B., Cardoner, N., Benet, O., ... & Harrison, B. J. (2018). Fear extinction in the human brain: a meta-analysis of fMRI studies in healthy participants. *Neuroscience & Biobehavioral Reviews,* 88, 16-25.
